# Heartland Virus Infection in Elderly Patient Initially Suspected of Having Ehrlichiosis, North Carolina, USA

**DOI:** 10.3201/eid3012.240646

**Published:** 2024-12

**Authors:** Alexis M. Barbarin, Teresa G. Fisher, Michael H. Reiskind, Carl Williams, Bryan N. Ayres, Kristen L. Burkhalter, William L. Nicholson

**Affiliations:** North Carolina Division of Public Health, Raleigh, North Carolina, USA (A.M. Barbarin, T.G. Fisher, C. Williams); North Carolina State University, Raleigh (M.H. Reiskind); Centers for Disease Control and Prevention, Atlanta, Georgia, USA (B.N. Ayres, W.L. Nicholson); Centers for Disease Control and Prevention, Fort Collins, Colorado, USA (K.L. Burkhalter)

**Keywords:** ehrlichiosis, *Ehrlichia chaffeensis*, *Ehrlichia ewingii*, Heartland virus, Bourbon virus, co-infection, *Amblyomma americanum*, ticks, bacteria, vector-borne infections, viruses, zoonoses, North Carolina, United States

## Abstract

We report a patient in North Carolina, USA, with Heartland virus infection whose diagnosis was complicated by previous *Ehrlichia chaffeensis* infection. We identified *E. ewingii*–infected and Bourbon virus–infected tick pools at the patient’s residence. Healthcare providers should consider testing for tickborne viruses if ehrlichiosis is suspected.

*Ehrlichia chaffeensis* and *E. ewingii* are tickborne intracellular bacteria that cause human ehrlichiosis (*1*). In the United States, ehrlichiosis occurs primarily in south-central, southeastern, and mid-Atlantic states. In 2019, nearly half of *E. chaffeensis* ehrlichiosis cases occurred in 4 US states: Arkansas, Missouri, New York, and North Carolina (*2*). Heartland virus (HRTV), another tickborne pathogen, is an emerging zoonotic virus and has been reported in 14 states, including North Carolina, since its initial discovery in 2009 in Missouri. (*3*). We report a case of HRTV infection in a patient in North Carolina who was initially suspected of having ehrlichiosis. 

## The Study

In March 2022, a North Carolina resident sought care at an emergency room and was subsequently admitted to the hospital because of a suspected case of ehrlichiosis. The patient sought care after experiencing 48 hours of fever, chills, shortness of breath, and diarrhea. Upon admission, the patient had acute leukopenia, thrombocytopenia, anemia, acute kidney injury, transaminitis, abdominal distension with splenomegaly, and meningoencephalitis. Hospital staff discovered 2 ticks attached to the patient and identified them as lone star ticks (*Amblyomma americanum*). Identification by a North Carolina Division of Public Health (NCDPH) entomologist (A.M.B.) subsequently confirmed the ticks as male and nymphal lone star ticks; the ticks collected from the patient were not tested for pathogens. Although no history of travel was reported, the patient practiced multiple daily walks to the edge of their 5.6-acre property, located in rural North Carolina.

The patient was initially treated for tickborne pathogen infections and meningitis by using broad spectrum antimicrobial drugs, including doxycycline, on day 2 after admission. On day 3, the patient required increased support because of progressive encephalopathy, hypotension, lactate elevation, and concerns of gastrointestinal bleeding, along with thrombocytopenia and was transferred to the medical intensive care unit. The patient experienced continued fevers and altered mental status. Results of differential testing for other infectious etiologies were negative (Table 1). Laboratory results indicated secondary hemophagocytic lymphohistiocytosis (Appendix Table), an increasingly recognized complication of rickettsial diseases (*4*,*5*). The patient was placed on anakinra on day 8 after hospital admission to address the hemophagocytic lymphohistiocytosis, and mental status slowly improved. Although the patient experienced mild improvement after treatment with anakinra and doxycycline, it was decided that the patient should transition to home hospice care after 18 days in the hospital. The patient recovered after 4 weeks at home and was removed from hospice care.

**Table 1:** Vectorborne disease testing in case study of Heartland virus infection in elderly patient initially suspected of having ehrlichiosis, North Carolina, USA*

Test, specimen source	Specimen collection date	Result	Reference values
Vectorborne and zoonotic organisms			
***Ehrlichia chaffeensis* IgG, serum**	2022 Mar 29	**1:512 titer**	<1:64 titer
** Heartland virus real-time RT-PCR, serum**	2022 Apr 5	**Positive**	Negative
Bourbon virus real-time RT-PCR, serum	2022 Apr 5	Negative	Negative
Bourbon PRNT, serum	2022 Apr 5	<1:10 titer, negative	<1:10 titer
Heartland PRNT, serum	2022 Apr 5	<1:10 titer, negative	<1:10 titer
*Rickettsia rickettsii* IgG, serum	2022 Apr 5	1:64 titer	<1:128 titer
***E. chaffeensis* IgG, serum**	2022 Apr 5	**1:1,024 titer**	<1:64 titer
*R. rickettsii* IgG, serum	2022 May 11	1:64 titer	<1:128 titer
***E. chaffeensis* IgG, serum**	2022 May 11	**1:512 titer**	<1:64 titer
*E. chaffeensis* PCR, serum	2022 Mar 29	Negative	Negative
Eastern equine encephalitis virus IgM, serum	2022 Apr 2	Negative	Negative
Viruses			
Hepatitis A virus IgG, serum	2022 Mar 28	Positive	Negative
Hepatitis A virus IgM, serum	2022 Mar 28	Negative	Negative
Hepatitis B surface antibody, serum	2022 Mar 28	Negative	Negative
Hepatitis B core antibody, serum	2022 Mar 28	Negative	Negative
Hepatitis C virus antibody, serum	2022 Mar 28	Negative	Negative
Cytomegalovirus IgG, serum	2022 Mar 28	Positive	Negative
Cytomegalovirus IgM, serum	2022 Mar 28	Negative	Negative
Cytomegalovirus PCR, blood	2022 Apr 1	Negative	Negative
Herpes simplex virus 1/2 PCR, serum	2022 Apr 2	Negative	Negative
Herpes simplex virus 1 IgG, serum	2022 Apr 2	Positive	Negative
Herpes simplex virus 2 IgG, serum	2022 Apr 2	Negative	Negative
Epstein-Barr virus PCR, serum	2022 Mar 31	Negative	Negative
HIV-1/2 antibody, serum	2022 Mar 29	Negative	Negative
HIV-1 p24 antigen, serum	2022 Mar 29	Negative	Negative
Sapovirus PCR, feces	2022 Mar 29	Negative	Negative
Adenovirus F40/41 PCR, feces	2022 Mar 29	Negative	Negative
Astrovirus PCR, feces	2022 Mar 29	Negative	Negative
Norovirus PCR, feces	2022 Mar 29	Negative	Negative
Rotavirus A PCR, feces	2022 Mar 29	Negative	Negative
Respiratory syncytial virus PCR, nasopharyngeal swab	2022 Mar 27	Negative	Negative
Influenza A/B PCR, nasopharyngeal swab	2022 Mar 27	Negative	Negative
SARS-CoV-2 PCR, nasopharyngeal swab	2022 Mar 27	Negative	Negative
Parvovirus IgM, serum	2022 Mar 29	Negative	Negative
Bacteria			
*Streptococcus pneumoniae* antigen, urine	2022 Apr 2	Negative	Negative
*Legionella pneumophila* antigen, urine	2022 Mar 31	Negative	Negative
*Coxiella burnetii* PCR, serum	2022 Apr 2	Negative	Negative
*Campylobacter* PCR, feces	2022 Mar 29	Negative	Negative
*Plesiomonas shigelloides* PCR, feces	2022 Mar 29	Negative	Negative
*Salmonella* PCR, feces	2022 Mar 29	Negative	Negative
*Vibrio cholerae* PCR, feces	2022 Mar 29	Negative	Negative
*Vibrio* PCR, feces	2022 Mar 29	Negative	Negative
*Yersinia enterocolitica* PCR, feces	2022 Mar 29	Negative	Negative
Enteroaggregative *Escherichia coli* PCR, feces	2022 Mar 29	Negative	Negative
Enteropathogenic *E. coli* PCR, feces	2022 Mar 29	Negative	Negative
Enterotoxigenic *E. coli* PCR, feces	2022 Mar 29	Negative	Negative
Enteroinvasive *E. coli* PCR, feces	2022 Mar 29	Negative	Negative
Shiga-toxin producing *E. coli* PCR, feces	2022 Mar 29	Negative	Negative
*Clostridioides difficile* PCR, feces	2022 Mar 29	Negative	Negative
*Francisella tularensis*, serum	2022 Apr 2	Negative	Negative
Culture, blood	2022 Apr 5	No growth	No growth
Parasites			
*Cryptosporidium* PCR, feces	2022 Mar 29	Negative	Negative
*Cryptosporidium cayetanensis* PCR, feces	2022 Mar 29	Negative	Negative
*Entamoeba histolytica* PCR, feces	2022 Mar 29	Negative	Negative
*Giardia lamblia* PCR, feces	2022 Mar 29	Negative	Negative
Parasite smear, feces	2022 Mar 31	Negative	Negative
Toxoplasma IgM/IgG, serum	2022 Apr 2	Negative	Negative

*Bold text indicates positive acute test result. PRNT, plaque reduction neutralization test; RT-PCR, reverse transcription PCR.

Indirect fluorescence antibody testing of serum revealed the patient was positive for *E. chaffeensis* IgG on day 4 after symptom onset, indicating a history of *Ehrlichia* infection (Table 1). Because of limited improvement after doxycycline administration and concerns about possible arbovirus infection, staff (T.G.F.) at NCDPH coordinated specimen collection with the local county health department to send a serum sample to the Centers for Disease Control and Prevention (CDC) in Fort Collins, Colorado, USA, for testing. Quantitative reverse transcription PCR (RT-PCR) was positive for HRTV RNA (*6*).

In conjunction with university partners, NCDPH conducted environmental sampling. In May and June 2022, we conducted standard tick drags at the patient’s home. The habitat surrounding the home was primarily deciduous, hardwood forest that had some understory growth and heavy leaf litter. We collected ticks by passing a 1-m^2^ cotton drag cloth over ground level vegetation. We checked the cloth every 10 m^2^ and collected ticks for pathogen testing (*7*). We collected 608 ticks in May and 656 ticks in June 2022 after 5 hours of total collection time (Table 2). We sent *A. americanum* tick specimens collected in May 2022 to CDC in Atlanta, Georgia, USA, and in Fort Collins to test for Bourbon virus (BRBV), HRTV, *E. chaffeensis*, and *E. ewingii*. We sent *A. americanum* ticks collected in June 2022 to CDC in Fort Collins for BRBV and HRTV testing. *E. chaffeensis* and *E. ewingii* DNA testing consisted of real-time PCR that amplified an 82-bp fragment of the 16S rRNA gene for both species and used 2 probes specific for either *E. chaffeensis* or *E. ewingii* detection (*8*). CDC tested ticks for HRTV and BRBV by using real-time RT-PCR primers and probes specific for each virus, as described previously (*9*,*10*). Pathogen testing of *A. americanum* ticks did not detect HRTV but did detect 2 *E. ewingii*–positive tick pools (1 pool = 25 nymphs; 1 pool = 5 adult ticks) out of a total of 43 pools (587 total ticks), indicating a minimum infection rate (MIR) of 0.34% (2 pools/587 ticks). In addition, BRBV was also detected in 1 tick pool (n = 25 ticks), indicating an MIR of 0.08% (1 pool/1,264 total ticks).

**Table 2:** Entomologic surveillance at patient’s residence in case study of Heartland virus infection in elderly patient initially suspected of having ehrlichiosis, North Carolina, USA

Surveillance data	May 2022	June 2022
Collection time	11 AM–2 PM	11 AM–1 PM
Mean air temperature, °F/°C	69/21	87/31
Area covered, m^2^	3,760	3,250
No. ticks collected, by species		
*Amblyomma americanum*, male	58	43
*A. americanum*, female	49	64
*A. americanum*, nymph	480	549
*A. maculatum*, nymph	1	0
*Dermacentor variabilis*, male	4	0
*D. variabilis*, female	2	0
*Ixodes scapularis*, male	9	0
*I. scapularis*, female	5	0
Total no. ticks collected	608	656

## Conclusions

This patient had a substantially elevated antibody titers against *E. chaffeensis* antigen at the time of symptom manifestation, suggesting a previous infection at some undetermined time; the lack of a rise in titer during hospitalization and the ineffectiveness of doxycycline argued against a current *Ehrlichia* infection. Therefore, other tickborne illnesses were considered for testing. The patient’s laboratory and clinical history were consistent with both HRTV and *Ehrlichia* spp. infections, but serologic and molecular testing are needed to distinguish between the 2 infections. The patient was negative for BRBV RNA by RT-PCR and negative for both HRTV and BRBV antibodies by using plaque reduction neutralization tests. However, additional testing for HRTV RNA revealed a positive RT-PCR result, indicating the patient had an active HRTV infection.

First identified in 2 farmers in Missouri, USA, in 2009, HRTV has been identified in >60 patients across the United States (*11*–*13*), including 1 previous case in North Carolina. In North Carolina, co-infection with *Ehrlichia* spp. and HRTV is possible because those pathogens are naturally maintained in a common tick vector. Although no evidence of co-infection existed in this case, the patient was likely exposed to both pathogens on their property. The additional findings of BRBV and *E. ewingii* in the collected tick pools indicate a further environmental risk for tickborne diseases at that residential site.

The MIR for *E. ewingii* in the collected ticks from this study is not uncommon for this region; prevalence rates for *Ehrlichia* spp. have been documented across the mid-Atlantic region. *E. chaffeensis* was detected in 2.6% and *E. ewingii* in 0.8% of *A. americanum* ticks in Tennessee (*14*). Virginia reported infection rates of 0%–5.08% for *E. chaffeensis* and 0%–8.20% for *E. ewingii* in *A. americanum* ticks (*15*). BRBV was identified in 1 pool of ticks collected in a North Carolina location where no BRBV in ticks has been previously reported. The presence of BRBV in *A. americanum* ticks indicates that citizens are at risk, albeit low, of contracting BRBV in North Carolina if bitten by lone star ticks.

Initial acute clinical features of ehrlichiosis and tickborne virus infections are similar. Therefore, healthcare providers should also consider testing patients for tickborne viruses if ehrlichiosis is suspected, especially when the infection is not responsive to doxycycline treatment. Furthermore, persons at risk for tick bites should use tick exposure prevention methods, such as applying N,N-diethyl-meta-toluamide and other US Environmental Protection Agency–approved repellants; wearing permethrin-treated clothing, long pants, and long sleeves; and performing tick checks after spending time in wooded areas.

Appendix Additional information for Heartland virus infection in elderly patient initially suspected of having ehrlichiosis, North Carolina, USA.
