# Supplementary material for: Heartland Virus Infection in Elderly Patient Initially Suspected of Having Ehrlichiosis, North Carolina, USA
Source: Emerg Infect Dis. 2024 Dec;30(12):2625–8. doi: 10.3201/eid3012.240646 (PMC11616643; doi:10.3201/eid3012.240646)
Supplement: Appendix — Additional information for Heartland virus infection in elderly patient initially suspected of having ehrlichiosis, North Carolina, USA. [file 24-0646-Techapp-s1.pdf]

Article DOI: https://doi.org/10.3201/eid3012.240646
EID cannot ensure accessibility for supplementary materials supplied by authors. Readers who have difficulty accessing supplementary content should contact the authors for assistance.

Heartland Virus Infection in Elderly Resident Initially Suspected of Having Ehrlichiosis, North Carolina, USA

Appendix

Appendix Table. Laboratory tests performed for North Carolina, USA, resident who had a tickborne disease*

Test	Reference range	Days after hospital admission 0	1	2	3	4	5	6	7	8	9	10	11	12	13	14	16
Body temperature, °C	36.5–37.2	37.1	38.8	39.3	38.3	39.4	37.2	NA	NA	NA	NA	NA	NA	NA	NA	36.7	36.5
Blood cell counts																	
Leukocytes, × 10^3 cells/µL	3.7–9.6	1.5	1.5	1.4	1.4	2	4.1	3.4	1.2	2.4	2.5	2.8	2.6	2.5	2.1	2.7	2.1
Hemoglobin, g/dL	12–17	12.7	10.8	11.5	11.6	11.9	11	8.7	9	8.6	8.7	7.7	7.3	8.3	8	8.4	7.5
Platelets, × 10^3/µL	123–309	69	58	49	34	34	16	27	17	13	29	25	25	37	42	46	33
Blood chemistry test results																	
Sodium, mmol/L	134–144	135	135	133	133	130	NA	NA	NA	140	NA	NA	141	144	144	142	142
Potassium, mmol/L	3.5–5.4	4.2	4	3.9	1.4	4.3	NA	NA	NA	4.3	NA	NA	3.7	3.5	3.5	3.7	4.2
Carbon dioxide, mmol/L	22–31	24	23	21	24	20	NA	NA	NA	19	NA	NA	24	25	25	24	24
Creatinine, mg/dL	0.6–1.4	2	1.7	2.2	2	1.8	NA	NA	NA	2.1	NA	NA	1.6	1.5	1.3	1.4	1.2
AST, IU/L	10–40	19	79	NA	94	147	NA	NA	NA	176	NA	NA	55	NA	NA	35	25
ALT, IU/L	10–60	41	89	NA	95	102	NA	NA	NA	93	NA	NA	67	NA	NA	58	43
ALP, IU/L	35–120	68	62	NA	55	53	NA	NA	NA	68	NA	NA	52	NA	NA	55	55
Other test results																	
D-dimer, mg/L	0–0.56	NA	NA	1.69	NA	NA	>20	>20	>20	NA	NA	11.03	10.66	NA	NA	NA	3.85
Lactic acid, mmol/L	0.4–2.0	1.9	4.2	NA	NA	4.3	4.1	4.2	3.6	NA	NA	NA	NA	NA	NA	NA	NA
Ferritin, ng/mL	30–400	NA	465	NA	NA	NA	NA	11,878.9	13,689.3	NA	4,015.8	2,140.9	1,428.1	1,232.3	883.1	766.4	536.2
Fibrinogen, mg/dL	213–541	NA	NA	352	NA	NA	281	254	233	NA	NA	161	147	153	NA	NA	156
CRP, mg/dL	0.02–0.7	NA	NA	NA	NA	2.62	NA	NA	NA	NA	NA	NA	NA	NA	NA	NA	0.38

*ALP, alkaline phosphatase; ALT, alanine aminotransferase; AST, aspartate aminotransferase; CRP, C-reative protein; NA, not applicable.
